# Supplementary material for: Absolute and relative reliability of pain sensitivity and functional outcomes of the affected shoulder among women with pain after breast cancer treatment
Source: PLoS One. 2020 Jun 3;15(6):e0234118. doi: 10.1371/journal.pone.0234118 (PMC7269234; doi:10.1371/journal.pone.0234118)
Supplement: S1 Appendix — (DOCX) [file pone.0234118.s001.docx]

| Check list of items - Guidelines for Reporting Reliability and Agreement Studies (GRRAS). | | Page No |
| --- | --- | --- |
| TITLE AND ABSTRACT | 1. The abstract identifies that intra-rater reliability was investigated. | P2, line 21. |
| INTRODUCTION | 2. The measurement devices and the experimental procedures are mentioned explicitly. | P6, line 119 to P10, Line 209 |
|  | 3. The subject population is specified. | P4, line 80-81 |
|  | 4. Not applicable (information on rater population). | NA |
|  | 5. Information about reliability and rationale for the study are provided. | P4, line 69-81 |
| METHODS | 6. We explain how the sample size was chosen. | P5, line 87-90 |
|  | 7. We explain the sampling method. | P5, line 91-99 |
|  | 8. We describe the measurement process including time interval between repeated measurements and availability of clinical information. | P6, line 109-112  S2 Appendix |
|  | 9. The measurements were made over two experimental sessions. | P 6, Line 110 and Figure 1 |
|  | 10. The statistical analysis is described. | P10-11, Line 211-234 |
| RESULTS | 11. We state the actual number of rater (one) and the included subjects as well as the number of replicate measurements . | P6, Line 114-115, P5, Line 90 and P6, Line 111-112 |
|  | 12. We describe the sample characteristics of the subjects. | S2 Appendix |
|  | 13. We report estimates of reliability and agreement including measures of statistical uncertainty. | P11, line 235 to P18, Line 318 |
| DISCUSSION | 14. We discuss the practical relevance of results. | P21, Line 383-386 |
| AUXILIARY MATERIAL | 15. We provide supplementary materials | S3 to S6 appendix |
