# Supplementary material for: Absolute and relative reliability of pain sensitivity and functional outcomes of the affected shoulder among women with pain after breast cancer treatment
Source: PLoS One. 2020 Jun 3;15(6):e0234118. doi: 10.1371/journal.pone.0234118 (PMC7269234; doi:10.1371/journal.pone.0234118)
Supplement: S2 Appendix — (DOCX) [file pone.0234118.s002.docx]

**TABLE 1.1:** SOCIODEMOGRAPHIC AND PHYSICAL PROFILE

| **CHARACTERISTIC**: | **CASES (N = 21)** |
| --- | --- |
| **Age, mean (CI: 95%), y** | 57.4 (54;60.8) |
| **Height, mean (CI: 95%), cm** | 167.9 (165.5;170.2) |
| **Living arrangement, No. (%)** |  |
| Living with a partner | 17 (81) |
| Living alone | 3 (14) |
| Other | 1 (15) |
| **Education, No. (%)** |  |
| Short | 6 (26) |
| Medium | 14 (67) |
| Long | 1 (5) |
| Other | 0 (0) |
| **Employment, No. (%)** |  |
| Full time | 11 (52) |
| Part time | 3 (14) |
| Staying at home | 0 (0) |
| Retired | 6 (29) |
| Sick leave | 0 (0) |
| Other | 1 (5) |
| **Body mass index, mean (CI: 95%), kg/m^2^** | 27.6 (25.3;29.8) |
| **Body mass index, No. (%)** |  |
| ≤ 25 kg/m^2^ | 8 (38) |
| >25 - ≤30 kg/m^2^ | 6 (29) |
| >30 kg/m^2^ | 7 (33) |
| **Menopausal status, No. (%)** |  |
| Pre | 1 (5) |
| Peri | 4 (19) |
| Post | 16 (76) |

**Abbrevations:** 95% Confidence interval: CI: 95%

**TABLE 2:** HEALTH BEAVIOUR

| **CHARACTERISTIC**: | **CASES (N = 21)** |
| --- | --- |
| **Level of physical activity, No. (%)** |  |
| Low | 6 (29) |
| Moderate | 10 (48) |
| High | 5 (24) |
| **Smoking, No. (%)** |  |
| Current smoker | 1 (5) |
| Exsmoker | 11 (52) |
| Never smoker | 9 (43) |
| **Alcohol consumption** |  |
| No. units per week, mean (CI: 95%) | 3.1 (1.5;4.8) |
| None, No. (%) | 8 (38) |

**Abbrevations:** 95% Confidence interval: CI: 95%

**TABLE 3:** MEDICAL AND SURGICAL PROFILE

| **CHARACTERISTIC**: | **CASES (N = 21)** |
| --- | --- |
| **Histologic stage of malignancy, No. (%)** |  |
| I | 6 (29) |
| II | 11 (52) |
| III | 4 (19) |
| **Tumor diameter, mean (CI: 95%), mm** | 17.8 (14.1;21.5) |
| **Surgical protocol, No. (%)** |  |
| Breast conserving surgery | 17 (81) |
| Mastectomy | 4 (19) |
| **Lymph node protocol No. (%)** |  |
| Sentinel lymph node biopsy | 15 (71) |
| Axillary dissection | 2 (10) |
| Both | 4 (19) |
| **No. of lymph nodes dissected, mean (CI: 95%),** | 4.9 (3.1;6.7) |
| **Dominant limb affected, No. (%)** | 9 (43) |
| **Adjuvant treatment, No. (%)** |  |
| Chemotherapy | 1 (5) |
| Radiotherapy | 4 (19) |
| Both | 16 (76) |
| **Endocrine therapy, No. (%)** |  |
| Currently | 10 (48) |
| Ceased | 4 (19) |
| **Receptor status, No. (%)** |  |
| Estrogen positive | 14 (67) |
| HER2 positive | 6 (29) |
| **Time since treatment, mean (CI: 95%), months** | 66.1 (51.3;80.8) |

**Abbrevations:** 95% Confidence interval: CI: 95%
