# Supplementary figures and images for: Absolute and relative reliability of pain sensitivity and functional outcomes of the affected shoulder among women with pain after breast cancer treatment
Source: PLoS One. 2020 Jun 3;15(6):e0234118. doi: 10.1371/journal.pone.0234118 (PMC7269234; doi:10.1371/journal.pone.0234118)

**
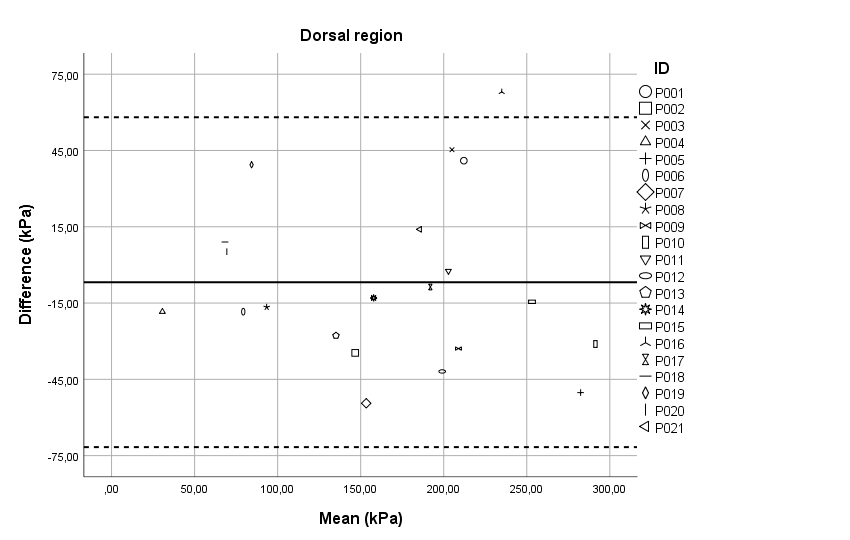
**

**
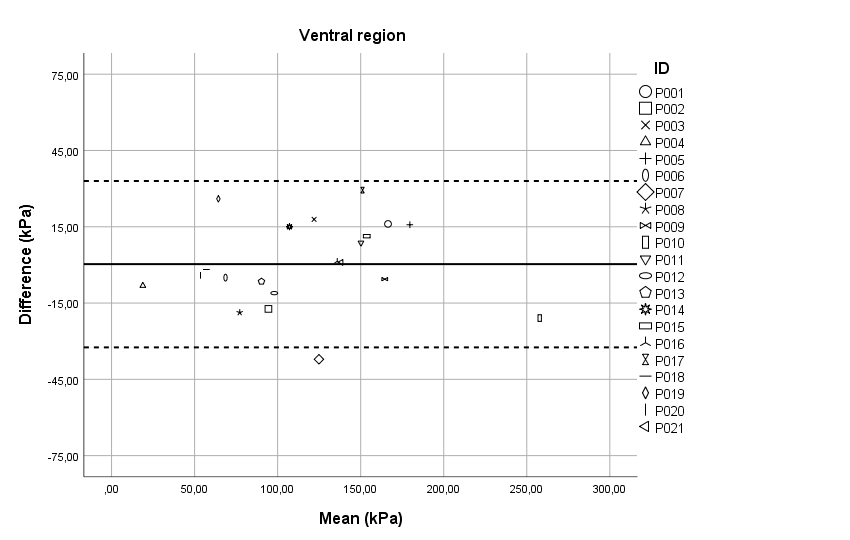
**


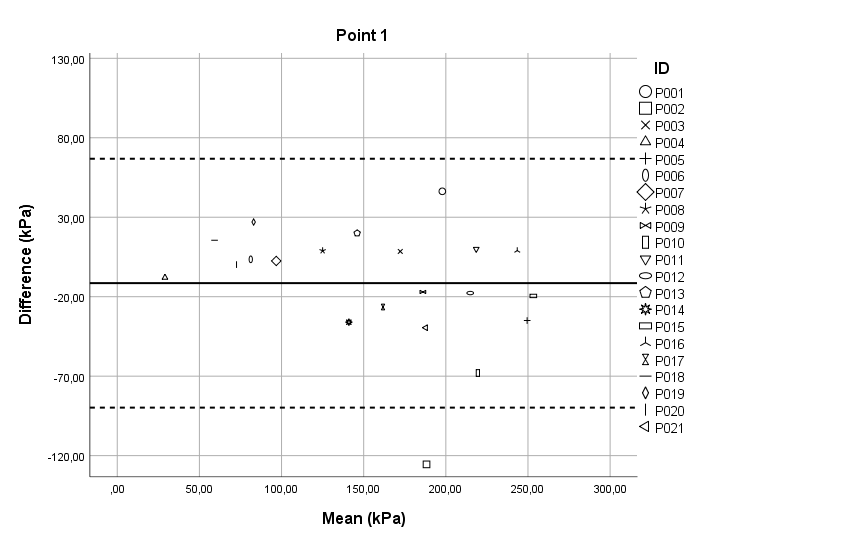

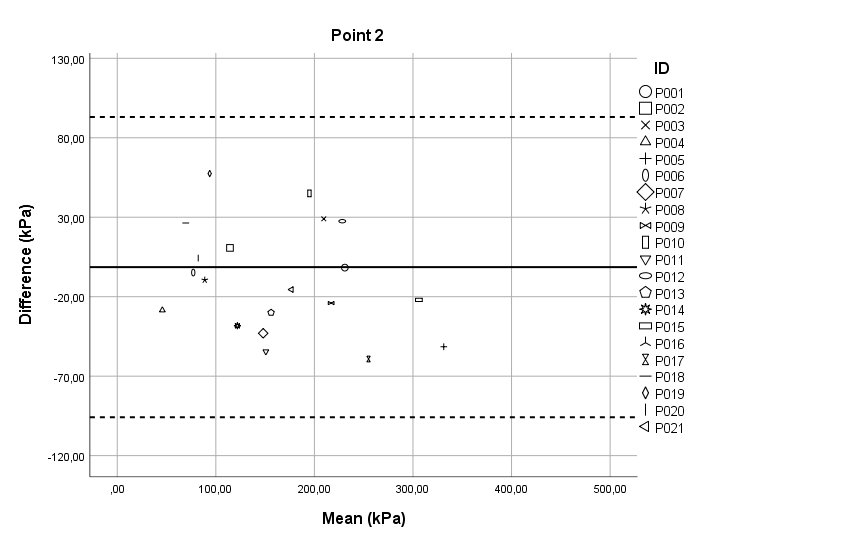

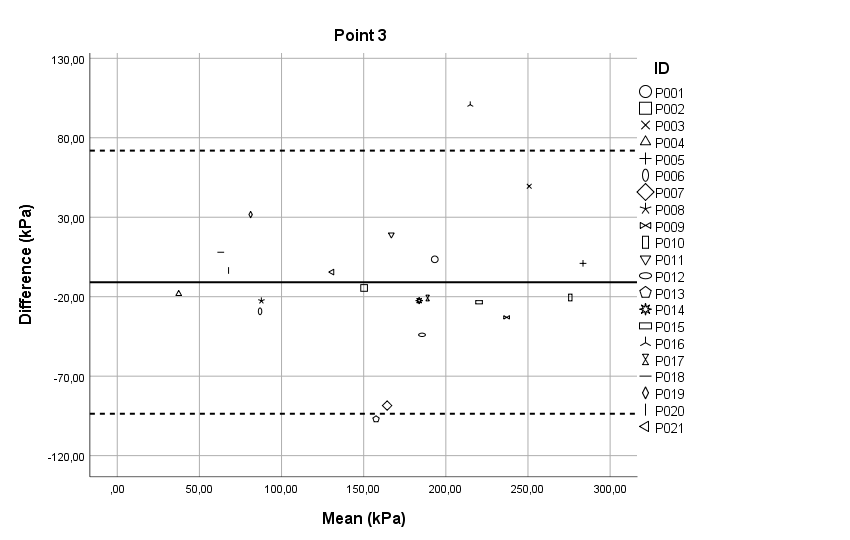

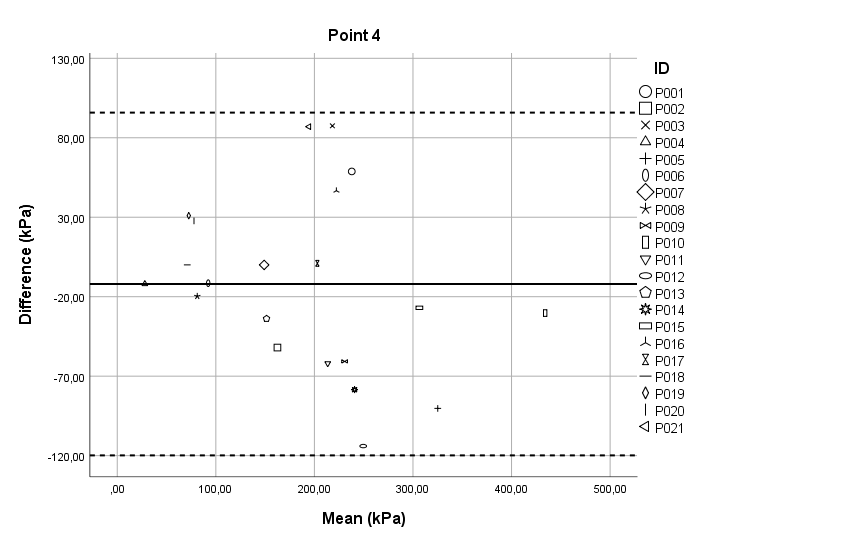

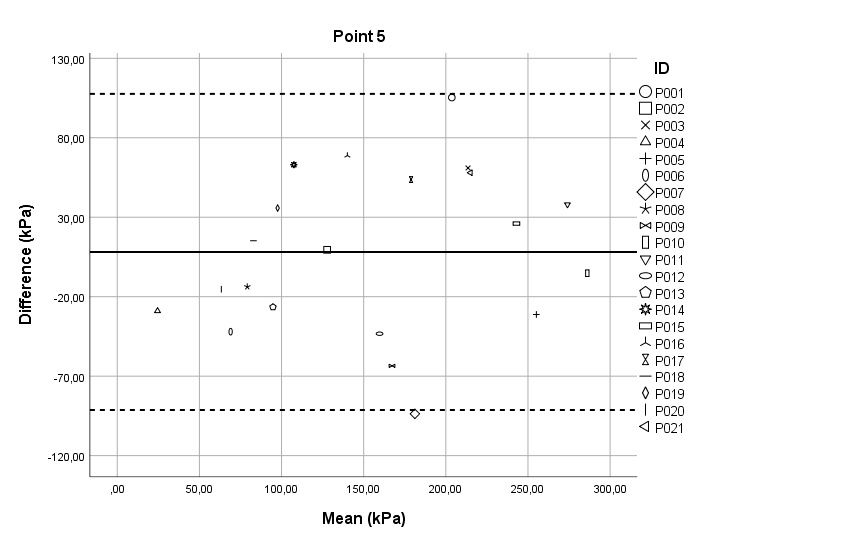

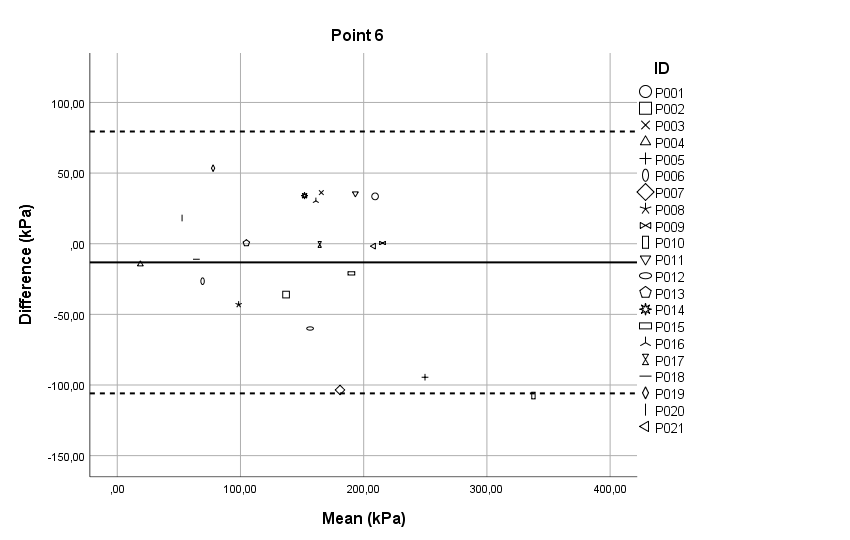

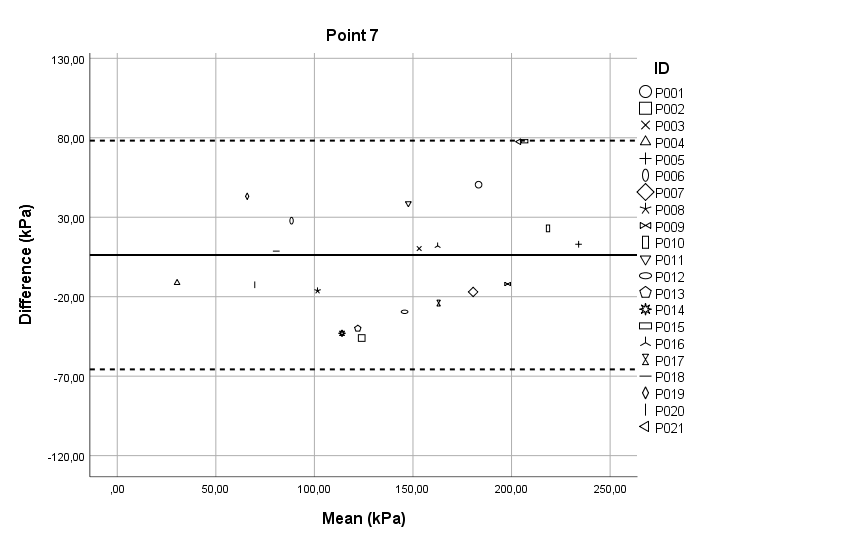

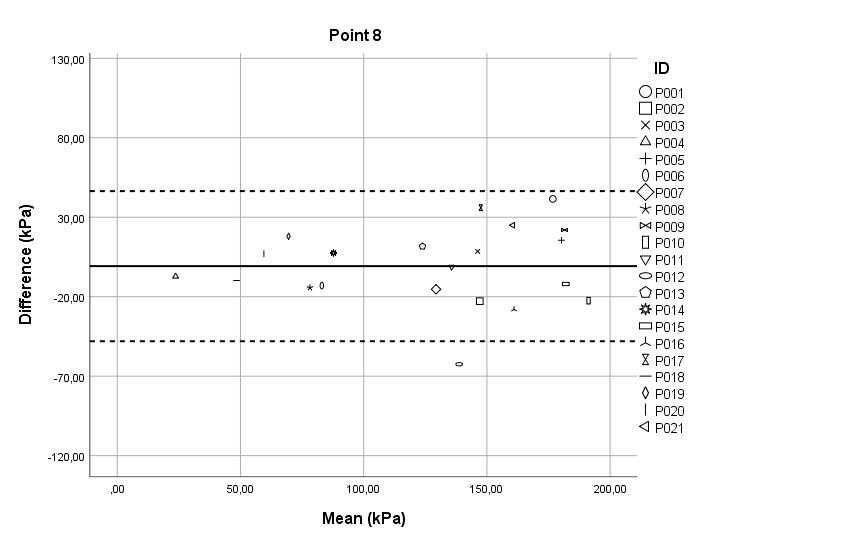

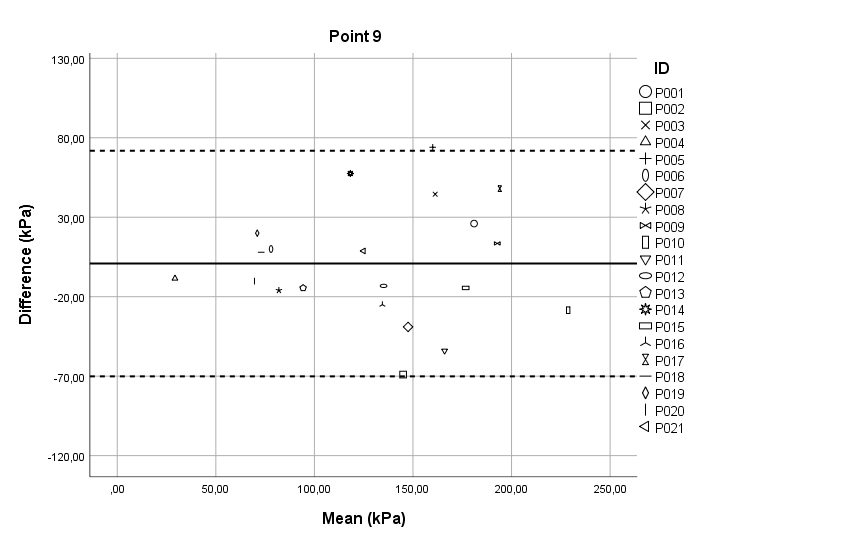

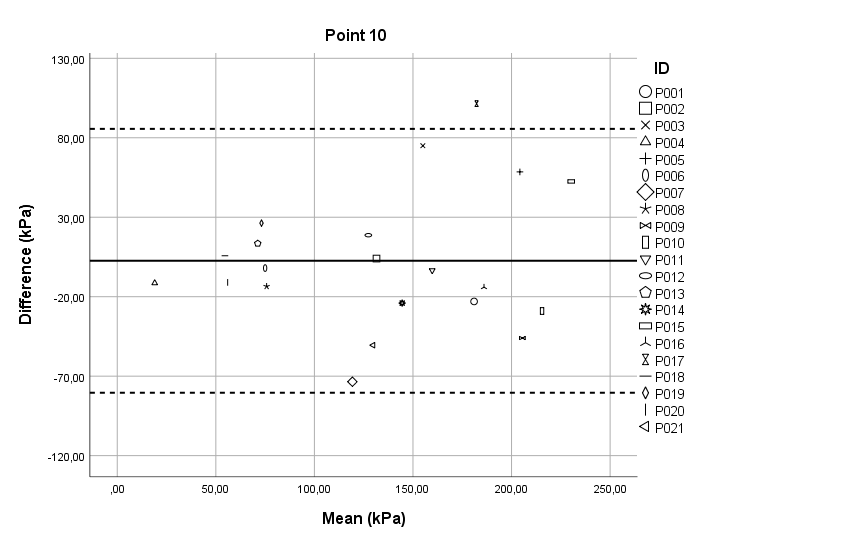

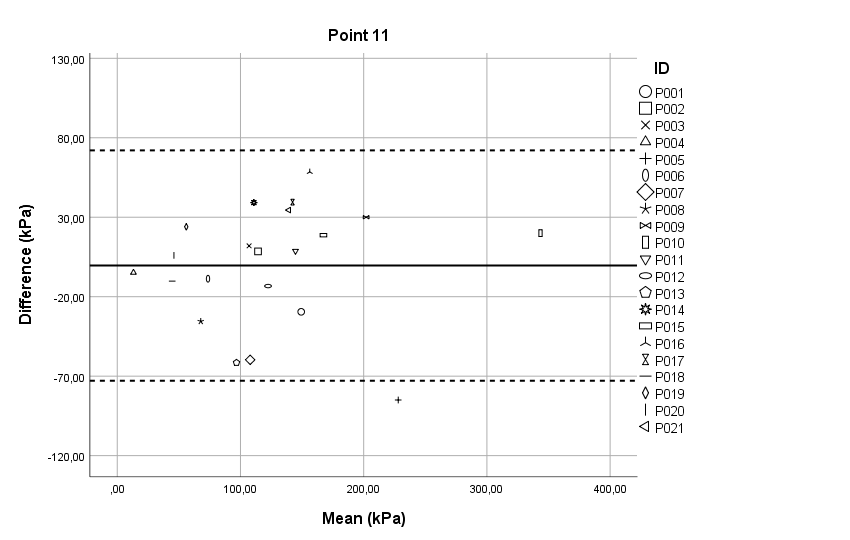

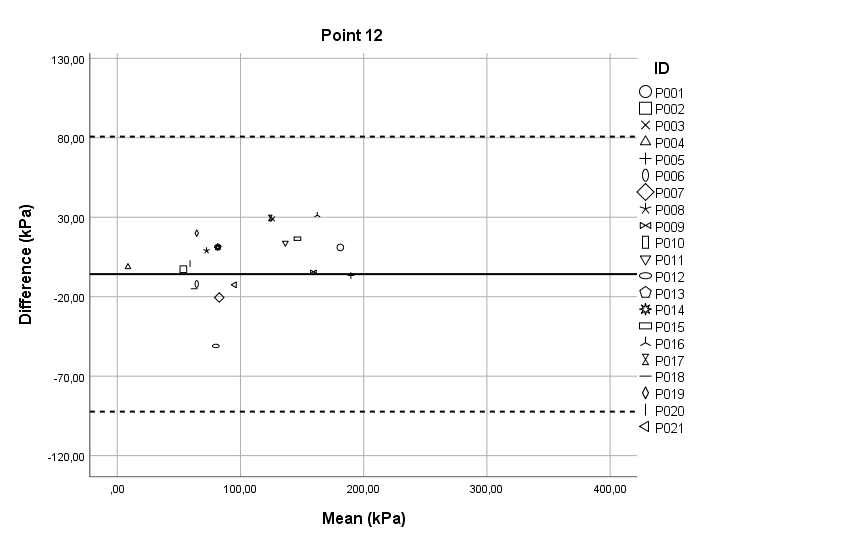

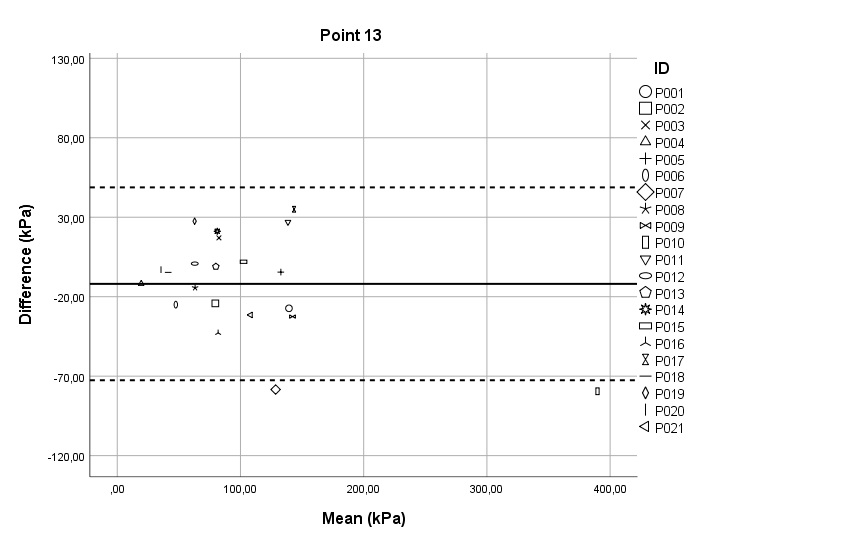

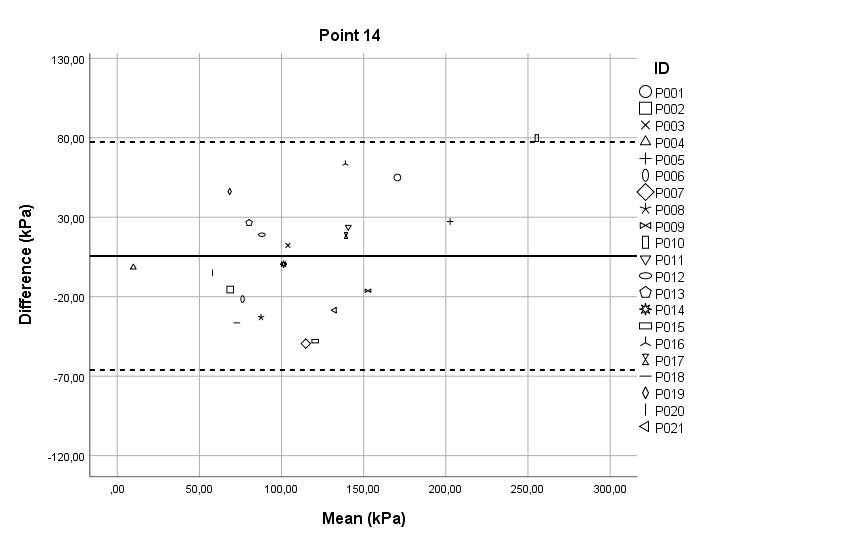

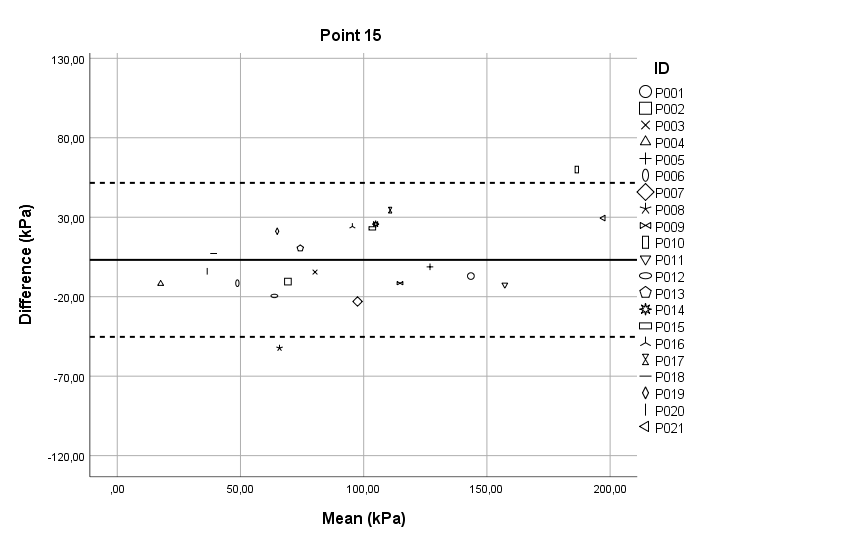

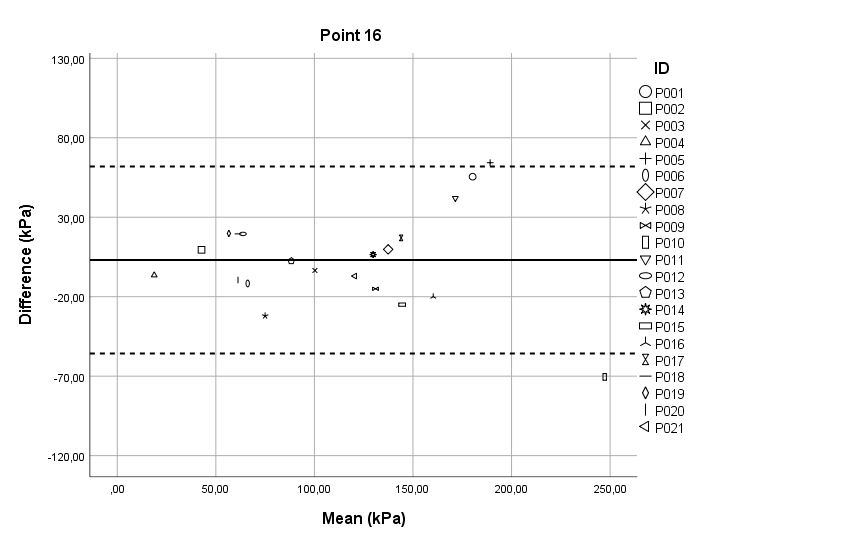

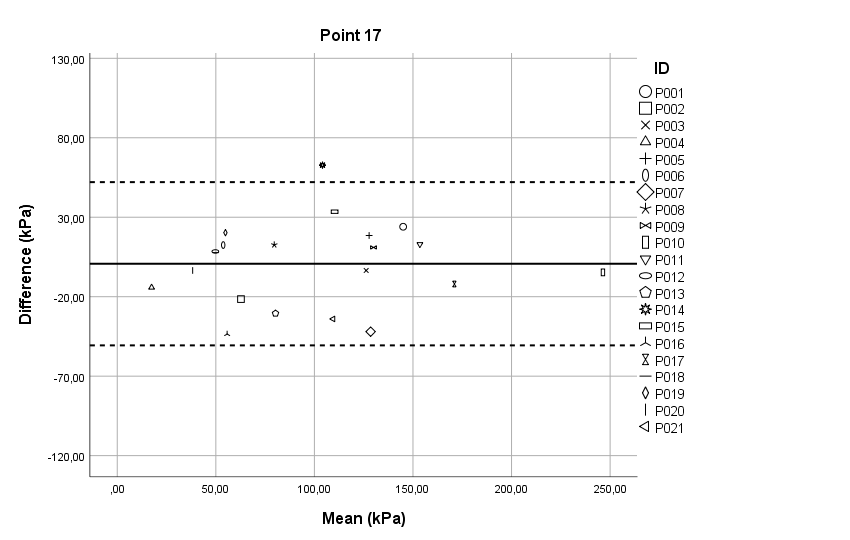

Supplement: S3 Appendix — (DOCX) [file pone.0234118.s003.docx]

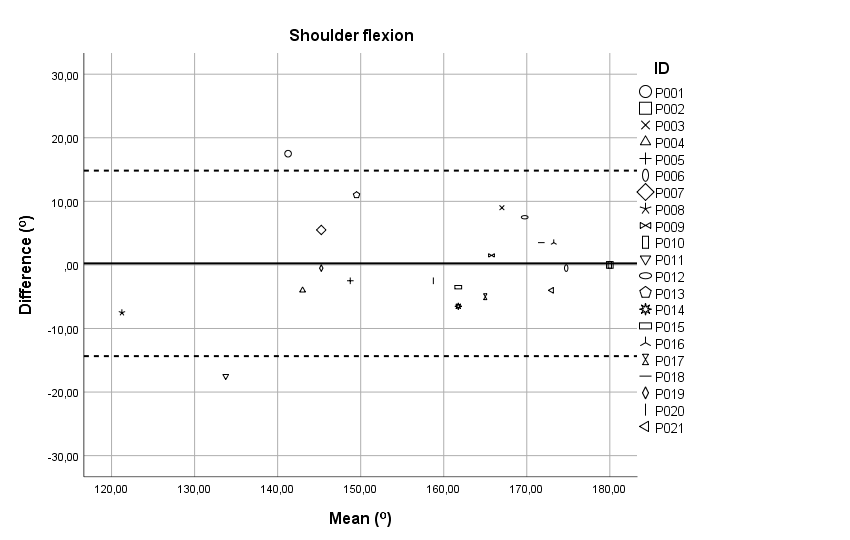

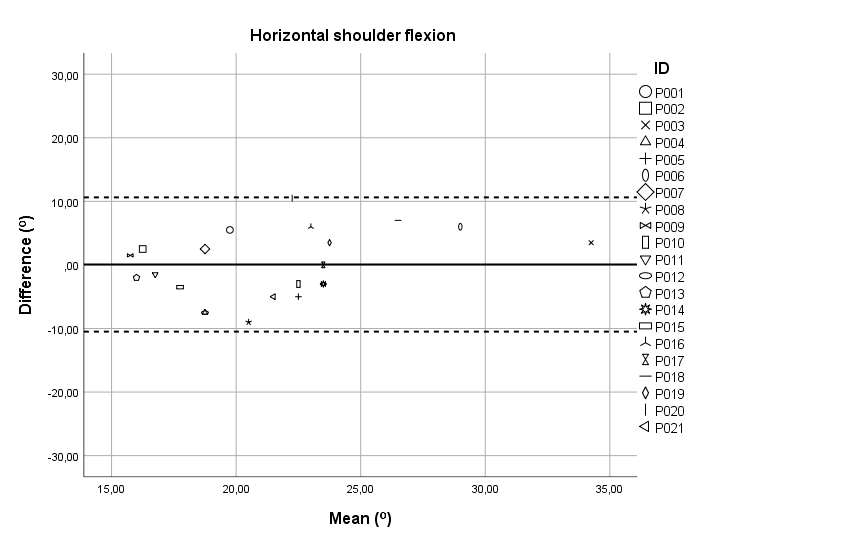

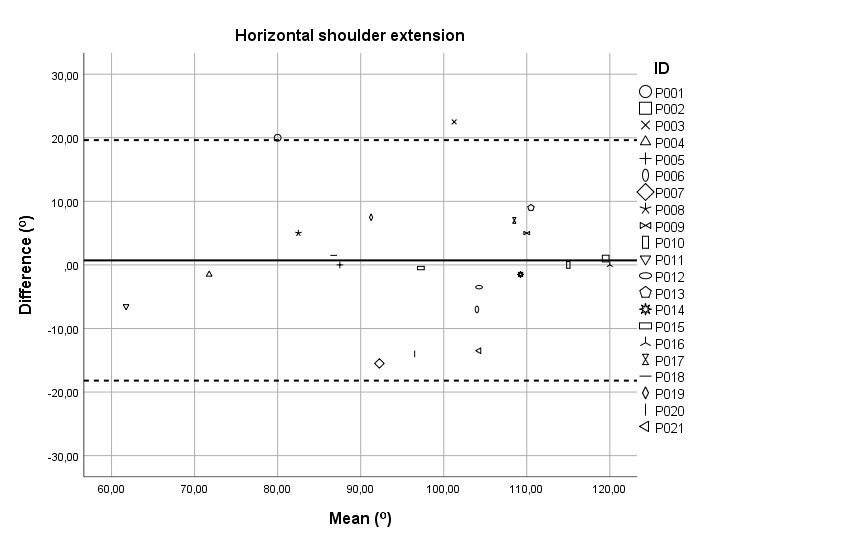

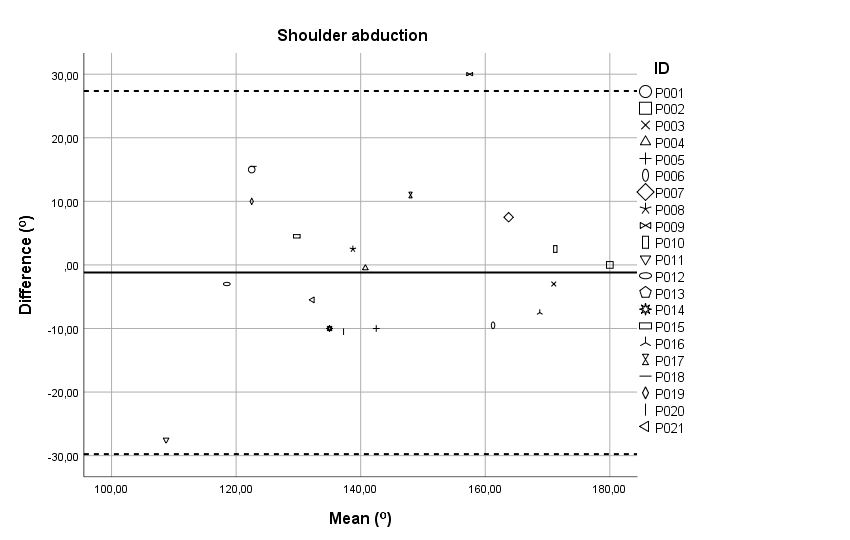

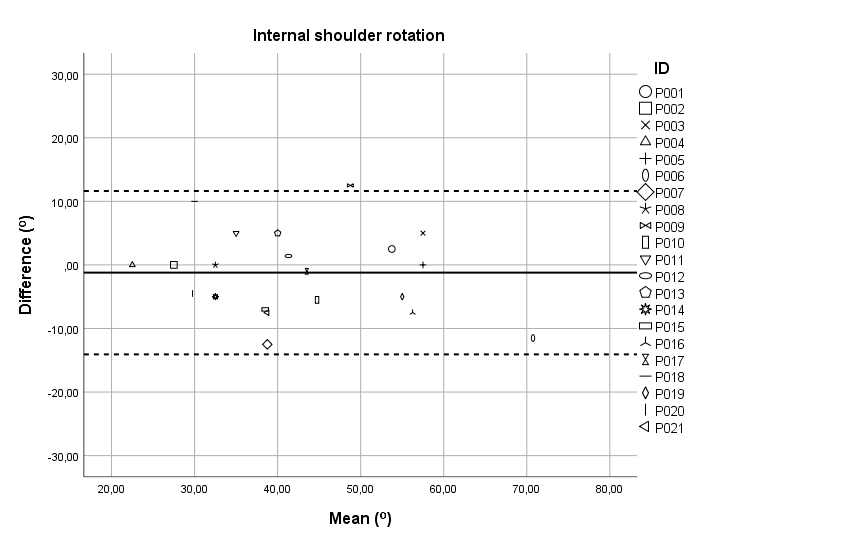

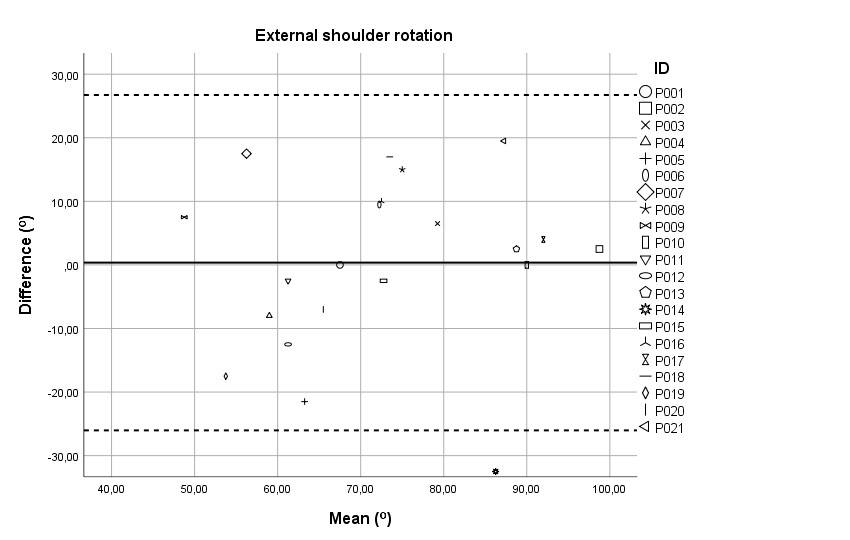

Supplement: S5 Appendix — (DOCX) [file pone.0234118.s005.docx]

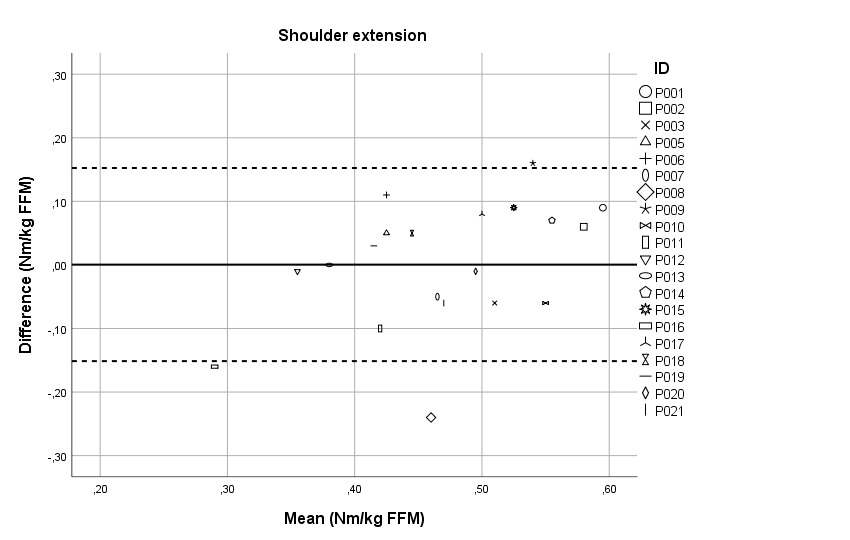

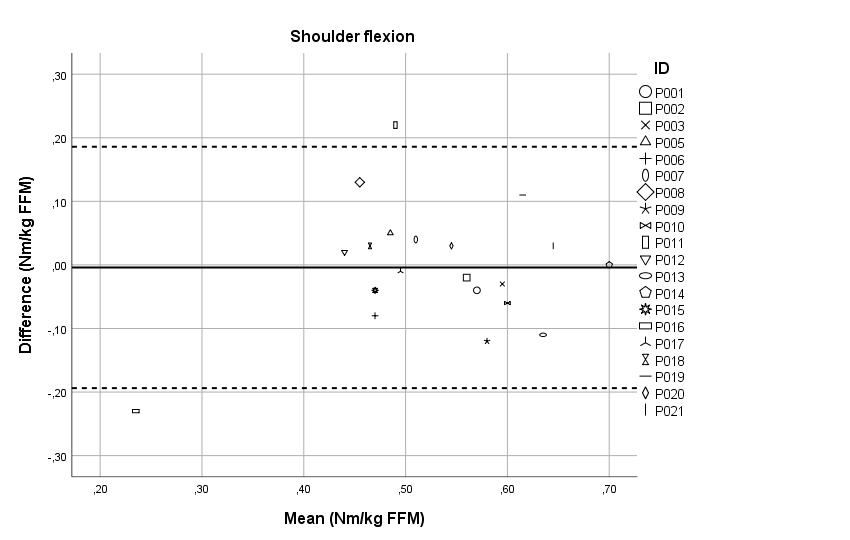

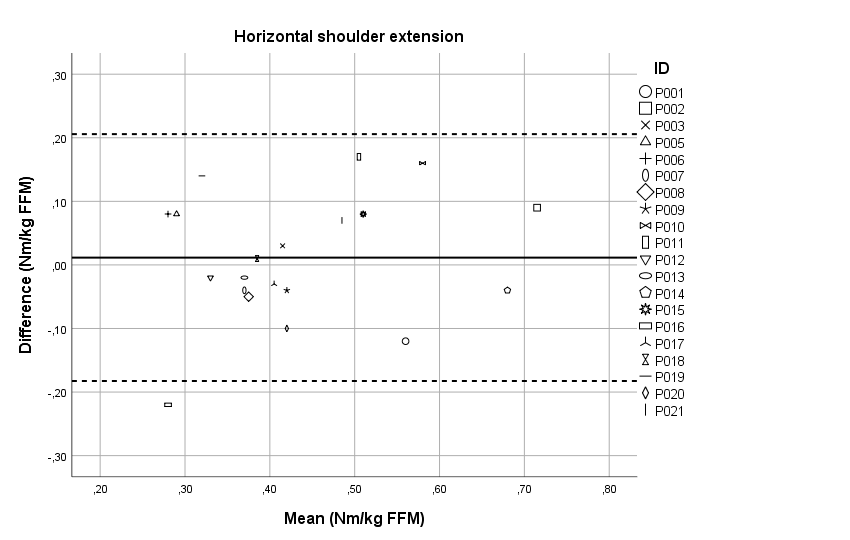

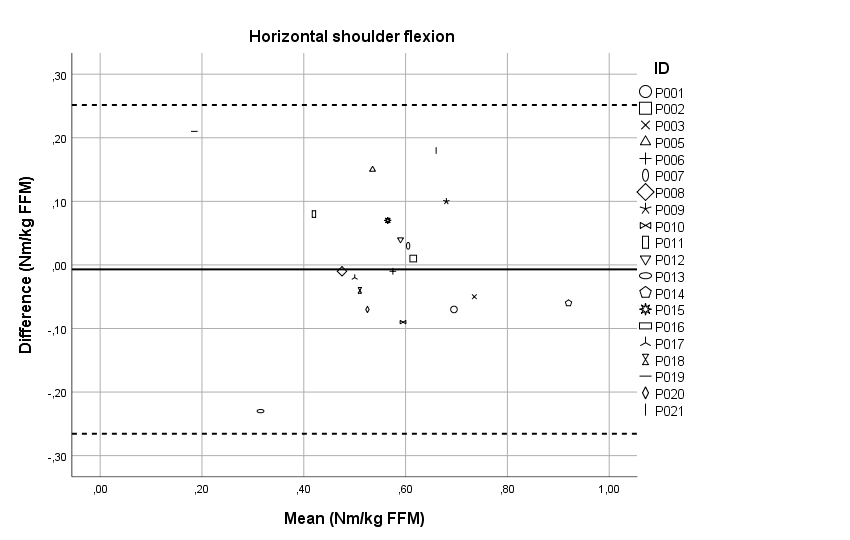

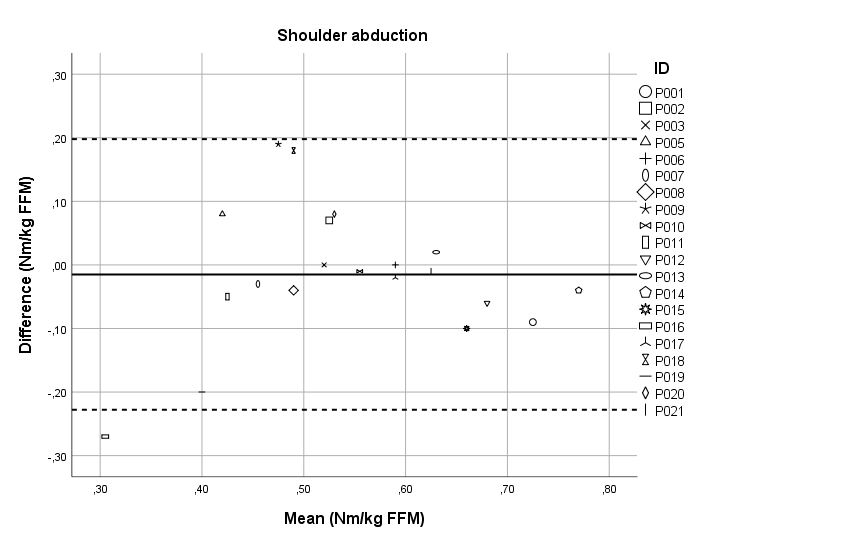

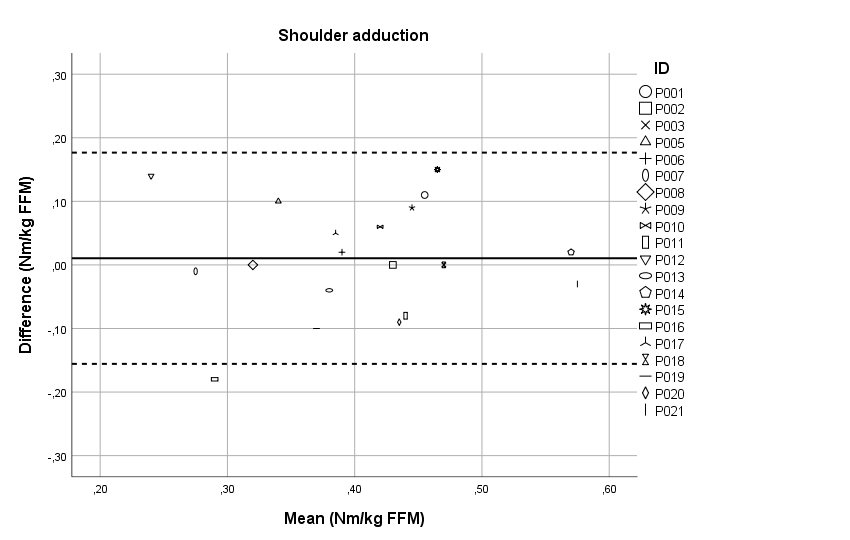

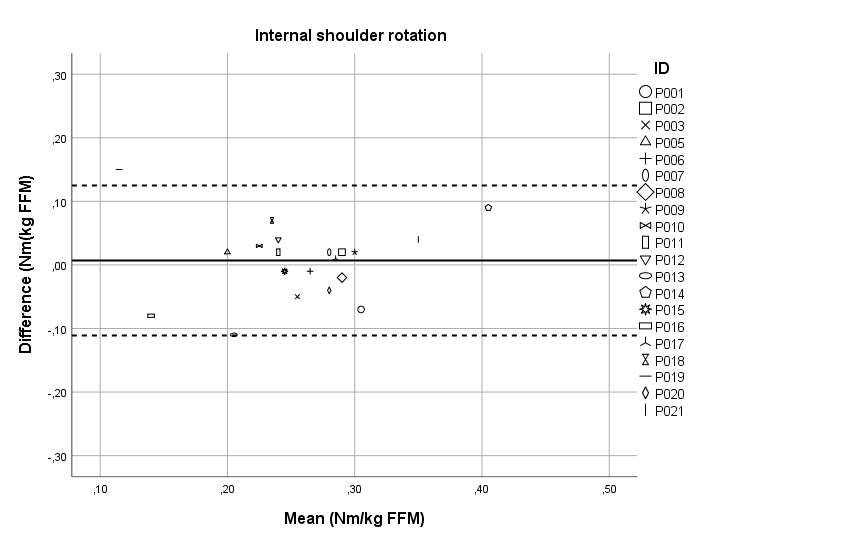

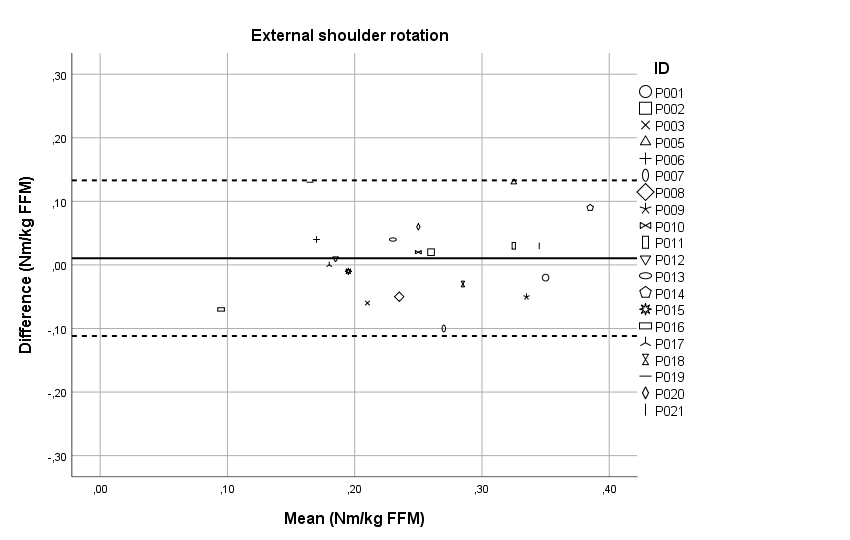

Supplement: S6 Appendix — (DOCX) [file pone.0234118.s006.docx]
