# Supplementary material for: Absolute and relative reliability of pain sensitivity and functional outcomes of the affected shoulder among women with pain after breast cancer treatment
Source: PLoS One. 2020 Jun 3;15(6):e0234118. doi: 10.1371/journal.pone.0234118 (PMC7269234; doi:10.1371/journal.pone.0234118)
Supplement: S4 Appendix — (DOCX) [file pone.0234118.s004.docx]

**Table 1:** Pairwise comparisons of pressure pain threshold for each location

| **PPT location** | **PPT location** | | | | | | | | | | | | | | | | | |
| --- | --- | --- | --- | --- | --- | --- | --- | --- | --- | --- | --- | --- | --- | --- | --- | --- | --- | --- |
|  |  | **P1** | **P2** | **P3** | **P4** | **P5** | **P6** | **P7** | **P8** | **P9** | **P10** | **P11** | **P12** | **P13** | **P14** | **P15** | **P16** | **P17** |
|  | **P1** | − | NS | NS | NS | NS | NS | NS | ↑ | NS | NS | NS | NS | NS | NS | NS | NS | NS |
|  | **P2** |  | − | NS | NS | NS | NS | NS | NS | NS | NS | NS | NS | NS | NS | NS | NS | NS |
|  | **P3** |  |  | − | NS | NS | NS | NS | ↑ | NS | ↑ | ↑ | ↑ | ↑ | ↑ | ↑ | ↑ | ↑ |
|  | **P4** |  |  |  | − | NS | NS | NS | ↑ | ↑ | ↑ | ↑ | ↑ | ↑ | ↑ | ↑ | ↑ | ↑ |
|  | **P5** |  |  |  |  | − | NS | NS | NS | NS | NS | NS | ↑ | NS | ↑ | ↑ | ↑ | ↑ |
|  | **P6** |  |  |  |  |  | − | NS | NS | NS | NS | ↑ | ↑ | ↑ | ↑ | ↑ | ↑ | ↑ |
|  | **P7** |  |  |  |  |  |  | − | NS | NS | NS | NS | NS | NS | ↑ | ↑ | NS | ↑ |
|  | **P8** |  |  |  |  |  |  |  | − | NS | NS | NS | NS | NS | NS | NS | NS |  |
|  | **P9** |  |  |  |  |  |  |  |  | − | NS | NS | NS | NS | NS | ↑ | NS | ↑ |
|  | **P1−** |  |  |  |  |  |  |  |  |  | − | NS | NS | NS | NS | NS | NS | NS |
|  | **P11** |  |  |  |  |  |  |  |  |  |  | − | NS | NS | NS | NS | NS | NS |
|  | **P12** |  |  |  |  |  |  |  |  |  |  |  | − | NS | NS | NS | NS | NS |
|  | **P13** |  |  |  |  |  |  |  |  |  |  |  |  | − | NS | NS | NS | NS |
|  | **P14** |  |  |  |  |  |  |  |  |  |  |  |  |  | − | NS | NS | NS |
|  | **P15** |  |  |  |  |  |  |  |  |  |  |  |  |  |  | − | NS | NS |
|  | **P16** |  |  |  |  |  |  |  |  |  |  |  |  |  |  |  | − | NS |
|  | **P17** |  |  |  |  |  |  |  |  |  |  |  |  |  |  |  |  | − |

Abbreviations: Pressure pain threshold: PPT. Non-significant: NS.

**↑**: P<0.05 (post-hoc analyses: PPT significantly higher).

**Table 2:** Pairwise comparisons of range of motion for each movement direction

| **Movement direction - ROM** | **Movement direction - ROM** | | | | | | |
| --- | --- | --- | --- | --- | --- | --- | --- |
|  |  | **Flexion** | **Horizontal flexion** | **Horizontal extension** | **Seated abduction** | **Internal rotation** | **External rotation** |
|  | **Flexion** | − | ↑ | ↑ | ↑ | ↑ | ↑ |
|  | **Horizontal flexion** |  | − | ↓ | ↓ | ↓ | ↓ |
|  | **Horizontal extension** |  |  | − | ↓ | ↑ | ↑ |
|  | **Seated abduction** |  |  |  | − | ↑ | ↑ |
|  | **Internal rotation** |  |  |  |  | − | ↓ |
|  | **External rotation** |  |  |  |  |  | − |

Abbreviations: Range of motion: ROM.

**↑**: P<0.001 (post-hoc analyses: ROM significantly higher).

**↓**: P<0.001 (post-hoc analyses: ROM significantly lower).

**Table 3:** Pairwise comparisons of maximal isokinetic muscle strength for each movement direction

| **Movement direction - MIMS** | **Movement direction - MIMS** | | | | | | | | |
| --- | --- | --- | --- | --- | --- | --- | --- | --- | --- |
|  |  | **Extension** | **Flexion** | **Horizontal flexion** | **Horizontal extension** | **Seated abduction** | **Seated adduction** | **Internal rotation** | **External rotation** |
|  | **Extension** | − | NS | NS | NS | NS | ↑ | ↑ | ↑ |
|  | **Flexion** |  | − | ↑ | NS | NS | ↑ | ↑ | ↑ |
|  | **Horizontal flexion** |  |  | − | ↑ | ↑ | NS | ↑ | ↑ |
|  | **Horizontal extension** |  |  |  | − | NS | ↑ | ↑ | ↑ |
|  | **Seated abduction** |  |  |  |  | − | ↑ | ↑ | ↑ |
|  | **Seated adduction** |  |  |  |  |  | − | ↑ | ↑ |
|  | **Internal rotation** |  |  |  |  |  |  | − | NS |
|  | **External rotation** |  |  |  |  |  |  |  | − |

Abbreviations: Maximal isokinetic muscle strength: MIMS.

**↑**: P<0.05 (post-hoc analyzes: MIMS significantly higher).
